# Supplementary material for: Pyruvate Dehydrogenase Kinase 1 inhibition mediated oxidative phosphorylation enhancement in cartilage promotes osteoarthritis progression
Source: BMC Musculoskelet Disord. 2023 Jul 20;24:597. doi: 10.1186/s12891-023-06585-6 (PMC10357736; doi:10.1186/s12891-023-06585-6)
Supplement: Supplementary file 1 — Additional file 1. [file 12891_2023_6585_MOESM1_ESM.zip › MF.Gsea.1653627316242/GOMF_CALCIUM_DEPENDENT_PROTEIN_KINASE_ACTIVITY.html]

Details for gene set GOMF\_CALCIUM\_DEPENDENT\_PROTEIN\_KINASE\_ACTIVITY[GSEA]

|  || Dataset | 55457\_collapsed\_to\_symbols.cls.cls #OA\_versus\_normal.cls.cls #OA\_versus\_normal\_repos |
| Phenotype | cls.cls#OA\_versus\_normal\_repos |
| Upregulated in class | normal |
| GeneSet | GOMF\_CALCIUM\_DEPENDENT\_PROTEIN\_KINASE\_ACTIVITY |
| Enrichment Score (ES) | -0.43164966 |
| Normalized Enrichment Score (NES) | -1.3838725 |
| Nominal p-value | 0.10296684 |
| FDR q-value | 0.28509527 |
| FWER p-Value | 1.0 |
Table: GSEA Results Summary

  

Fig 1: Enrichment plot: GOMF\_CALCIUM\_DEPENDENT\_PROTEIN\_KINASE\_ACTIVITY      
 Profile of the Running ES Score & Positions of GeneSet Members on the Rank Ordered List

  

| SYMBOL | TITLE | RANK IN GENE LIST | RANK METRIC SCORE | RUNNING ES | CORE ENRICHMENT || 1 | PINK1 | PTEN induced kinase 1 [Source:HGNC Symbol;Acc:HGNC:14581] | 2097 | 0.350 | -0.1025 | No |
| 2 | PRKCD | protein kinase C delta [Source:HGNC Symbol;Acc:HGNC:9399] | 2237 | 0.335 | -0.0556 | No |
| 3 | PKN1 | protein kinase N1 [Source:HGNC Symbol;Acc:HGNC:9405] | 2540 | 0.298 | -0.0276 | No |
| 4 | PRKCZ | protein kinase C zeta [Source:HGNC Symbol;Acc:HGNC:9412] | 2760 | 0.274 | 0.0026 | No |
| 5 | MAPKAPK3 | MAPK activated protein kinase 3 [Source:HGNC Symbol;Acc:HGNC:6888] | 3483 | 0.204 | -0.0183 | No |
| 6 | PRKCQ | protein kinase C theta [Source:HGNC Symbol;Acc:HGNC:9410] | 3658 | 0.189 | 0.0008 | No |
| 7 | CAMK4 | calcium/calmodulin dependent protein kinase IV [Source:HGNC Symbol;Acc:HGNC:1464] | 3664 | 0.189 | 0.0330 | No |
| 8 | PRKCA | protein kinase C alpha [Source:HGNC Symbol;Acc:HGNC:9393] | 3728 | 0.183 | 0.0597 | No |
| 9 | PRKD1 | protein kinase D1 [Source:HGNC Symbol;Acc:HGNC:9407] | 4123 | 0.144 | 0.0538 | No |
| 10 | MKNK1 | MAPK interacting serine/threonine kinase 1 [Source:HGNC Symbol;Acc:HGNC:7110] | 5830 | 0.007 | -0.0775 | No |
| 11 | PRKCE | protein kinase C epsilon [Source:HGNC Symbol;Acc:HGNC:9401] | 5926 | 0.000 | -0.0848 | No |
| 12 | MAPKAPK5 | MAPK activated protein kinase 5 [Source:HGNC Symbol;Acc:HGNC:6889] | 6094 | -0.012 | -0.0957 | No |
| 13 | PRKCB | protein kinase C beta [Source:HGNC Symbol;Acc:HGNC:9395] | 6453 | -0.039 | -0.1168 | No |
| 14 | PRKD3 | protein kinase D3 [Source:HGNC Symbol;Acc:HGNC:9408] | 6717 | -0.057 | -0.1275 | No |
| 15 | PRKD2 | protein kinase D2 [Source:HGNC Symbol;Acc:HGNC:17293] | 7011 | -0.081 | -0.1363 | No |
| 16 | PRKCH | protein kinase C eta [Source:HGNC Symbol;Acc:HGNC:9403] | 7141 | -0.092 | -0.1305 | No |
| 17 | PRKCG | protein kinase C gamma [Source:HGNC Symbol;Acc:HGNC:9402] | 8582 | -0.200 | -0.2079 | No |
| 18 | CCL3 | C-C motif chemokine ligand 3 [Source:HGNC Symbol;Acc:HGNC:10627] | 11465 | -0.497 | -0.3459 | Yes |
| 19 | MAPKAPK2 | MAPK activated protein kinase 2 [Source:HGNC Symbol;Acc:HGNC:6887] | 12094 | -0.619 | -0.2880 | Yes |
| 20 | MKNK2 | MAPK interacting serine/threonine kinase 2 [Source:HGNC Symbol;Acc:HGNC:7111] | 12236 | -0.655 | -0.1861 | Yes |
| 21 | PKN2 | protein kinase N2 [Source:HGNC Symbol;Acc:HGNC:9406] | 12237 | -0.655 | -0.0731 | Yes |
| 22 | PRKCI | protein kinase C iota [Source:HGNC Symbol;Acc:HGNC:9404] | 12444 | -0.723 | 0.0354 | Yes |
Table: GSEA details [plain text format]

  

Fig 2: GOMF\_CALCIUM\_DEPENDENT\_PROTEIN\_KINASE\_ACTIVITY      
 Blue-Pink O' Gram in the Space of the Analyzed GeneSet

  

Fig 3: GOMF\_CALCIUM\_DEPENDENT\_PROTEIN\_KINASE\_ACTIVITY: Random ES distribution      
 Gene set null distribution of ES for **GOMF\_CALCIUM\_DEPENDENT\_PROTEIN\_KINASE\_ACTIVITY**
